# Ghrelin therapy mitigates bone marrow injury and splenocytopenia by sustaining circulating G-CSF and KC increases after irradiation combined with wound

**DOI:** 10.1186/s13578-018-0225-3

**Published:** 2018-04-05

**Authors:** Juliann G. Kiang, Marsha N. Anderson, Joan T. Smith

**Affiliations:** 1 Radiation Combined Injury Program, Armed Forces Radiobiology Research Institute, Bethesda, MD 20889 USA; 2 Department of Pharmacology and Molecular Therapeutics, Uniformed Services University of the Health Sciences, Bethesda, MD 20814 USA; 3 Department of Medicine, Uniformed Services University of the Health Sciences, Bethesda, MD 20814 USA

**Keywords:** Ionizing radiation, Skin wound, Ghrelin, Bone marrow, WBC, RBC, Spleen, G-CSF, KC, Caspase, Apoptosis

## Abstract

**Background:**

Radiation injury combined wound (CI) enhances acute radiation syndrome and subsequently mortality as compared to radiation injury alone (RI). We previously reported that ghrelin (a 28-amino-acid-peptide secreted from the stomach) treatment significantly increased a 30-day survival, mitigated hematopoietic death, circulating white blood cell (WBC) depletion and splenocytopenia and accelerated skin-wound healing on day 30 after CI. Herein, we aimed to study the ghrelin efficacy at early time points after CI.

**Methods:**

B6D2F1/J female mice were exposed to ^60^Co-γ-photon radiation at 9.5 Gy (LD_50/30_) followed by a 15% total-body-surface-area skin wound. Several endpoints were measured at 4–5 h, days 1, 3, 7 and 15.

**Results:**

Histological analysis of sternums on day 15 showed that CI induced more adipocytes and less megakaryocytes than RI. Bone marrow cell counts from femurs also indicated CI resulted in lower bone marrow cell counts on days 1, 7 and 15 than RI. Ghrelin treatment mitigated these CI-induced adverse effects. RI and CI decreased WBCs within 4–5 h and continued to decrease to day 15. Ghrelin treatment mitigated decreases in CI mice, mainly from all types of WBCs, but not RBCs, hemoglobin levels and hematocrit values. Ghrelin mitigated the CI-induced thrombocytopenia and splenocytopenia. CI increased granulocyte-colony stimulating factor (G-CSF) and keratinocyte chemoattractant (KC) in blood and bone marrow. Ghrelin therapy was able to enhance and sustain the increases in serum on day 15, probably contributed by spleen and ileum, suggesting the correlation between G-CSF and KC increases and the neutropenia mitigation. Activated caspase-3 levels in bone marrow cells were significantly mitigated by ghrelin therapy on days 3 and 15.

**Conclusions:**

Our novel results are the first to suggest that ghrelin therapy effectively decreases hematopoietic death and splenocytopenia by sustaining circulating G-CSF and KC increases after CI. These results demonstrate efficacy of ghrelin as a radio-mitigator/therapy agent for CI.

## Background

Radiation exposure accidents and detonation in the past have shown that irradiated victims experienced radiation injury (RI) also often concurrently received other trauma such as wounds, burns, blast or hemorrhage as well, namely combined injury (CI). CI was observed after Atomic bombing, Hiroshima and Nagasaki, Japan and at the Chernobyl reactor meltdown with 60–70% of victims [1, 2] and 10% of victims [3], respectively received thermal burns concurrent with radiation injury. Burns, wounds and infections usually increase mortality after otherwise non-lethal radiation dose observed in animal models of CI including mice [4–12], rats [13–15], guinea pigs [16], dogs [17, 18] and swine [19]. CI also delays wound closure times from normally 14 days after wounding without radiation to more than 30 days after irradiation [4, 7, 8, 20].

CI results in much severe outcomes than RI. Accelerated body-weight loss, amplified cytokine and chemokine imbalance, systemic bacterial infection [4], enhanced leukocytopenia, thrombocytopenia and erythrocytopenia [5, 7, 8], acute myelosuppression [4], immune system inhibition, fluid imbalance, macro- and microcirculation failure, massive cellular damage and disruption of vital organ functions are observed and subsequently lead to multiple organ dysfunction (MOD) and multiple organ failure (MOF). As a result, death after CI occurs [16, 21, 22]. In our mouse model of RI and CI, the median lethal dose to cause 50% population dead over 30 days (LD_50/30_) for RI is around 9.65 Gy, whereas radiation-wound CI (R-W CI) is around 8.95 Gy. The dose modifying factor (DMF) is 1.08 [4]. It is speculated that intervention of CI-induced aggravation of body-weight loss, cytokine imbalance or bacterial sepsis should improve the survival. Drugs or biologics such as pegylated G-CSF [7], ciprofloxacin [8, 20], mesenchymal stem cells [23], and ghrelin [24], prove to be effective to increase 30-day survival. However, their underlying mechanisms are not elucidated yet. Ghrelin effects at early time points after CI in bone marrow histopathology, circulating blood cells, cytokines and chemokines, and apoptosis were investigated.

Among of them, ghrelin was studied. Ghrelin is a hormone that is a hunger-stimulating peptide containing 28 amino acids [25]. It is produced mainly by P/D1 cells lining the fundus of the human stomach and epsilon cells of the pancreas [26]. Ghrelin levels increase before meals and decrease after meals. It’s counterpart hormone is leptin produced by adipose tissue [27].

Ghrelin potently stimulates growth hormone from the anterior pituitary gland [25]. Ghrelin activates the endothelial isoform of nitric oxide synthase (eNOS) in a pathway [28] that depends on PI3K/Akt/eNOS/NO signal pathway [29, 30]. Ghrelin binds on growth hormone secretagogue receptors that is coupled to G-protein [25].

It is reported that human ghrelin decreased organ injury and increased survival by 30% above the vehicle-treated mice after RI combined with severe sepsis in rats [14]. Kiang et al. [24] reported that ghrelin therapy was efficacious in a mouse model of radiation combined with wound or burn by increasing survival, body weight, wound healing, bone marrow cell counts, neutrophil recovery and platelet recovery on day 30 post CI as well as inhibiting brain hemorrhage [31]. Ghrelin blocks NF-κB activation, decreased TNF-α and IL-6 concentrations in lung of septic rats and inhibited nucleotide-binding oligomerization domain-containing protein 2 [NOD2, also known as caspase recruitment domain-containing protein 15 (CARD15) or inflammatory bowel disease protein 1 (IBD1)] [32]. NOD2 is important for apoptosis and the NF-κB activation pathways [33]. Ghrelin inhibits IκB and increases Th1 cytokine and IL-17 secretion in primary T cells [34]. Because RI and CI induce increased production of inflammatory cytokines and chemokines [4–6], it is thought that interventions which would early mitigate inflammatory responses could likely improve survival after CI. We, therefore, wanted to elucidate the dynamic changes in hematopoietic cell death and its related signaling molecules such as granulocyte-colony stimulating factor (G-CSF) and keratinocyte chemattractant (KC) at early time points. This report provides evidence from an experimental CI animal model, which was designed to demonstrate a kinetic change in hematopoiesis in circulating blood cells correlating with circulating G-CSF and KC and bone marrow G-CSF, KC, and caspase-3 activity.

## Methods

### Experimental design

B6D2F1/J female mice were randomly divided into 8 groups: (1) sham vehicle, (2) wound vehicle, (3) RI vehicle, (4) CI vehicle, (5) sham ghrelin, (6) wound ghrelin, (7) RI ghrelin, and (8) CI ghrelin. Groups 2, 4, 6 and 8 received topical gentamicin cream; groups 1–8 were administered with oral levofloxacin. N = 6 mice per group per time point. Hematological analysis, spleen weights, splenocyte counts, sternum histopathology and femur bone marrow cell counts of surviving animals were performed at each specified time point. The AFRRI Institutional Animal Care and Use Committee reviewed and approved all animal procedures. Euthanasia was carried out in accordance with the recommendations and guidance of the American Veterinary Medical Association [35, 36].

### Animals

B6D2F1/J female mice (The Jackson Laboratory, Bar Harbor, ME) were maintained in a facility accredited by the Association for Assessment and Accreditation of Laboratory Animal Care International in plastic microisolator cages on hardwood chip bedding. Commercial rodent chow and acidified tap water were provided ad libitum at 12–20 weeks of age. Animal holding rooms were maintained at 21 ± 1 °C with 50 ± 10% relative humidity using at least 10 changes/h of 100% conditioned fresh air. A 12-h 0600 (light) to 1800 (dark) full-spectrum lighting cycle was used.

### Gamma irradiation

Mice were given 9.5 Gy [LD_50/30_, bilateral, see Ref. 5] whole-body bilateral ^60^Co γ-photon radiation, delivered at a dose rate of 0.4 Gy/min, while held in vertically stacked, ventilated, four-compartment, acrylic plastic boxes that provided electron equilibrium during irradiation. Empty compartments within the boxes were filled with 3-inch-long × 1-inch-diameter acrylic phantoms to ensure uniform electron scattering. The mapping of the radiation field was performed with alanine/EPR dosimetry [37] using standard alanine calibration sets from US National Institute of Standards and Technology and National Physical Laboratory of the United Kingdom. The mapping provided dose rates to water measured by alanine pellets placed in the hollowed core of the acrylic phantoms in each compartment of the mouse rack on the day of the mapping. The field was uniform within ± 1.8% over all the 120 compartments. The exposure time for each irradiation was determined from the mapping data; corrections for the ^60^Co decay and the small difference in the mass energy absorption coefficients for water and soft tissue were applied. The accuracy of the actual dose delivery was verified with an ionization chamber adjacent to the mouse rack, which had been calibrated in terms of dose to the mid-line soft tissue of mice.

### Skin injury

Skin surface injuries were performed on the shaved dorsal surface of mice. Animals receiving skin wounds were anesthetized by isoflurane inhalation. A 15% total body-surface-area skin wound was performed within 1 h after irradiation [4]. All mice subjected to the skin injury were given 0.5 mL sterile 0.9% NaCl intraperitoneally (i.p.), which contained 150 mg/kg of acetaminophen (AmerisourceBergen, Glen Alen, Virginia), immediately after skin injury to alleviate pain. The sham group received the same handling procedure except administration of 0.5 ml sterile 0.9% NaCl containing 150 mg/kg of acetaminophen.

### Ghrelin

Ghrelin was purchased from Phoenix Pharmaceutical (Burlingame, CA). Three doses of 113 µg/kg were administered by lateral tail-vein injections [14] in a volume of 0.2 ml 24, 48 and 72 h after RI or CI. The vehicle given to control mice was sterile 0.9% sodium chloride solution for injection, USP.

### Antimicrobial agents

Gentamicin sulfate cream, 0.1% (generic, E. Fougera and Co., Melville, N.Y., NDC 0168-007-15), was applied daily for 10 days to the skin injuries on days 1–10. Levofloxacin (LVX), (generic, Aurobindo Pharma, Ltd., Mahaboob Nagar, India, NDC 65862-537-50), 100 mg/kg in 0.2 ml/mouse, was administered *p.o*. daily for 14 days on days 3–16. Briefly, a 500-mg tablet was crushed by mortar and pestle. The LVX in the powder was dissolved in a volume of sterile water approximately one-third the total volume required to prepare the concentration needed for the average body mass of the mice to be treated. The mortar was rinsed with the remaining two-thirds volume of sterile water. The combined suspension was centrifuged to remove the particulate filler and the supernatant solution was passed through a 0.45-µm membrane filter into a sterile amber bottle, which was sealed with a sterile rubber stopper and stored at 4–8 °C [7].

### Assessment of blood cell profile in peripheral blood

Blood samples were collected in EDTA tubes on 4–5 h, days 1, 3, 7, and 15 after RI or CI (N = 6 mice per group per time point) and assessed with the ADVIA 2120 Hematology System (Siemens, Deerfield, IL). Differential analysis was conducted using the peroxidase method and the light scattering techniques recommended by the manufacturer.

### Measurements of spleen weights and splenocytes

Spleens were collected from each euthanized mouse on 4–5 h, days 1, 3, 7, and 15 after RI or CI (N = 6 mice per group per time point). Each specimen was weighed, placed in a plastic bag with 10 ml of 1× Hank’s Balanced Salt Solution (Invitrogen, Grand Island, NY) homogenized using Seward Stomacher^®^ 80 (Thermo Scientific), and poured through a 70 mm cell strainer (BD Falcon, Bedford, MA). The fluid with Splenocytes was then centrifuged at 800×*g* (Sorvall Legend XTR Centrifuge, Thermo Scientific) for 10 min. The pellet was resuspended in 10 mL 1× ACK lysis buffer (Invitrogen) for 10 min at 37 °C to lyse RBCs, mixed by vortexing, and then centrifuged at 800×*g*. Splenocytes were collected, resuspended in 10 mL 1× phosphate-buffered saline (PBS), placed in Countess™ cell-counting-chamber slides (Invitrogen, Eugene, Oregon) and counted using the Countess automated cell counter (Invitrogen).

### Measurements of bone marrow cells

Bone marrow cells from femurs were collected on 4–5 h, days 1, 3, 7, and 15 after RI or CI (N = 6 mice per group per time point) and washed with 10 ml 1× PBS. The cells were then centrifuged at 800×*g*, re-suspended in 10 ml 1× PBS buffer, placed in Countess™ cell-counting-chamber slides (Invitrogen) and counted using the Countess automated cell counter (Invitrogen).

### Histopathology assessment

Sternum specimens were collected from mice on day 15 (n = 6 mice per group). Specimens were rinsed in cold saline solution and immediately fixed in 10% phosphate-buffered formalin. The tissue was then embedded in paraffin, sectioned transversely and stained with H&E. The histology slides were scanned using Zeiss Axioscan.Z1. Then, adipocytes and megakaryocytes were counted [4] using Zen 2 software (Zeiss Company, Thornwood, NY).

### Measurement of G-CSF and KC

Blood samples were collected on 4–5 h, days 1, 3, 7, and 15 after RI or CI (N = 6 mice per group per time point) after RI or CI using BD Microtainers (Becton, Dickinson and Company, Franklin Lakes, NJ). Blood samples were placed at room temperature for 30 min and centrifuged at 9600×*g* for 10 min (Sovall Legend Micro 21 Centrifuge, Thermo Scientific). Then serum was collected. Spleen, ileum, bone marrow, and kidney were minced, blended with beads, homogenized with Bullet Blender Storm 24 (Averill Park, NY), and centrifuged at 9600×*g* for 10 min. The supernatants were collected. G-CSF and KC concentrations were measured and analyzed using the Bio-PlexTM Cytokine Assay (Bio-Rad; Hercules, CA) following the manufacturer’s directions. Briefly, serum samples and tissue lysates from each animal were diluted fourfold and examined in duplicate. Data were analyzed using the LuminexH 100TM System (Luminex Corp.; Austin, TX) and quantified using MiraiBio MasterPlexH CT and QT Software (Hitachi Software Engineering America Ltd.; San Francisco, CA), and concentrations were expressed in pg/mL unless otherwise noted. The cytokines analyzed were IL-1α IL-1β, IL-2, IL-3, IL-4, IL-5, IL-6, IL-9, IL-10, IL-12(p40), IL-12(p70), IL-13, IL-17, eotaxin, G-CSF, GM-CSF, IFN-γ, KC, MCP-1, MIP-1a, MIP-1b, RANTES and TNF-a. Data were expressed as pg/mL in serum and pg/mg protein in tissues [4]. G-CSF and KC Data were herein reported.

### Activated caspase-3 measurement

Activated caspase-3 protein levels were measured using Quantikine ELISA kit according to the manufacturer’s protocol (R&D SYSTEM, Minneapolis, MN).

### Statistical analysis

Parametric data are expressed as the mean ± sem. For each survival experiment, 6 mice per group were tested on an individual basis. One-way ANOVA, two-way ANOVA, studentized-range test, and Student’s t-test were used for comparison of groups, with 5% as a significant level.

## Results

### Ghrelin therapy increases bone marrow cellularity

**Fig. 1:**
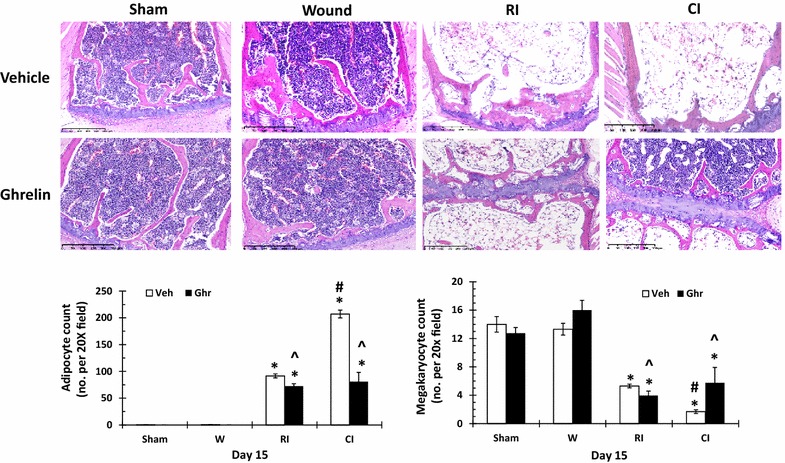
Ghrelin therapy increases bone marrow cellularity. Animals were irradiated alone or followed by wounding. Sternums on day 15 were collected for histology with H&E staining (N = 4 per group). Adipocytes and megakaryocytes were counted. *p < 0.05 vs. Sham group; ^^^p < 0.05 vs. respective Veh group; ^#^p < 0.05 vs. RI + Veh group. *Veh* vehicle, *Ghr* ghrelin, *W* wounding, *RI* 9.5 Gy, *CI* 9.5 Gy + wounding

It is evident that RI alone induced an increase in adipocyte cell count and CI further induced the increase [38]. Figure 1 shows that the similar outcome was observed. Ghrelin treatment mitigated the increases in both RI and CI mice.

It is also evident that RI alone induced a decrease in megakaryocyte cell counts and CI further enhanced the decrease [38]. Comparable results were obtained herein. Ghrelin therapy significantly recovered the cell count in CI mice but not in RI mice (Fig. 1). Wounding alone with vehicle or ghrelin treatment did not alter the bone marrow morphology.

### Ghrelin therapy increases bone marrow cell counts

**Fig. 2:**
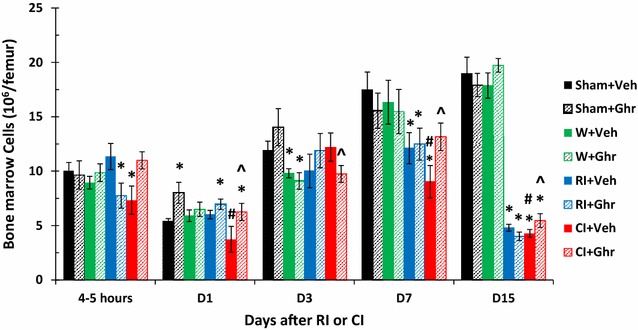
Ghrelin therapy increases bone marrow cell counts. Animals were irradiated alone or followed by wounding. Femurs on different time points were collected for measuring bone marrow cell counts (N = 6 per group). *p < 0.05 vs. Sham group; ^^^p < 0.05 vs. respective Veh group; ^#^p < 0.05 vs. RI + Veh group. *Veh* vehicle, *Ghr* ghrelin, *W* wounding, *RI* 9.5 Gy, *CI* 9.5 Gy + wounding

To confirm the RI-induced bone marrow cell depletion and the CI enhancement of bone marrow cell depletion, bone marrow cells were collected from femurs of each animal at different time points post RI and CI treated with vehicle or ghrelin. As shown in Fig. 2, RI significantly reduced bone marrow cell counts from femurs on days 7 and 15. CI enhanced the decreases at 4–5 h and days 1 and 7. Ghrelin treatment significantly recovered bone marrow counts at days 1, 7, and 15. Wounding alone with vehicle or ghrelin did not alter the counts.

### Ghrelin therapy recovers WBCs after wounding and CI

**Fig. 3:**
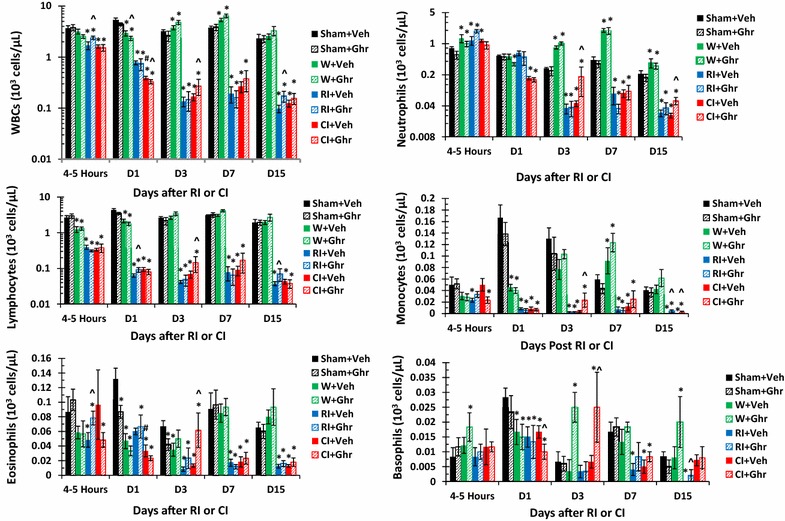
Ghrelin therapy recovers WBCs after wounding and CI. Animals were irradiated alone or followed by wounding. Blood on different time points were collected for measuring white blood cell counts (WBCs; N = 6 per group). *p < 0.05 vs. Sham group; ^^^p < 0.05 vs. respective Veh group; ^#^p < 0.05 vs. RI + Veh group. *Veh* vehicle, *Ghr* ghrelin, *W* wounding, *RI* 9.5 Gy, *CI* 9.5 Gy + wounding

It is reported that RI decreased WBCs and CI further decreased them [5]. Figure 3 shows that RI-induced decreases in WBCs began within 4–5 h, continued to decrease on day 1 and 3 and remained at the nadir on days 7 and 15. The decreases were contributed by neutrophils, lymphocytes, monocytes, eosinophils and basophils. CI further decreased the WBCs on day 1 due to the further reduction of neutrophils. Ghrelin therapy recovered WBCs on day 3. However, data from individual types of WBCs indicated that ghrelin therapy recovered neutrophils and monocytes on days 3 and 15 and lymphocytes, eosinophils and basophils on day 3. Wounding with ghrelin increased neutrophils and lymphocytes on days 3, 7, and 15, monocytes on days 7 and 15, and basophiles on days 3 and 15.

### Ghrelin therapy does not change RBCs after wounding, RI or CI

**Fig. 4:**
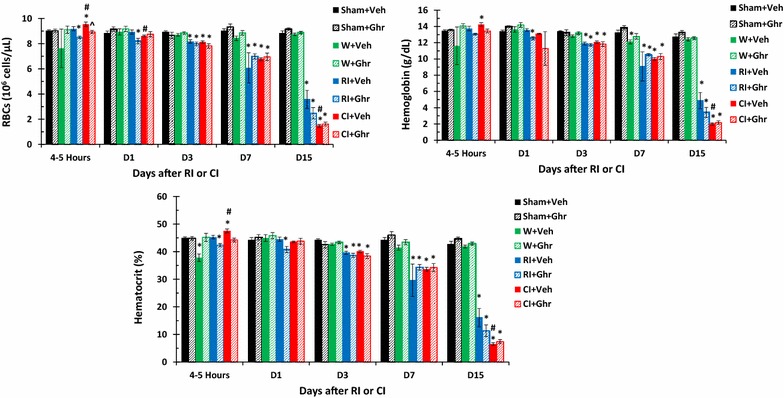
Ghrelin therapy does not change RBCs after wounding, RI or CI. Animals were irradiated alone or followed by wounding. Blood on different time points were collected for measuring red blood cell counts (RBCs), hemoglobin levels, and hematocrit values (N = 6 per group). *p < 0.05 vs. Sham group; ^#^p < 0.05 vs. RI + Veh group. *WBCs* white blood cells, *Veh* vehicle, *Ghr* ghrelin, *W* wounding, *RI* 9.5 Gy, *CI* 9.5 Gy + wounding

It is known that RI decreased RBCs, hemoglobin and hematocrit and CI further decreased them [5]. Figure 4 shows that RI-induced decreases in RBCs began on day 3 and continued to decrease on day 15. CI-induced enhancement was observed on day 15. Ghrelin therapy failed to recover RBCs. Similar observations were obtained with hemoglobin levels and hematocrit readings. Wounding alone with either vehicle or ghrelin did not alter these parameters.

### Ghrelin therapy increases platelet counts after wounding and CI

**Fig. 5:**
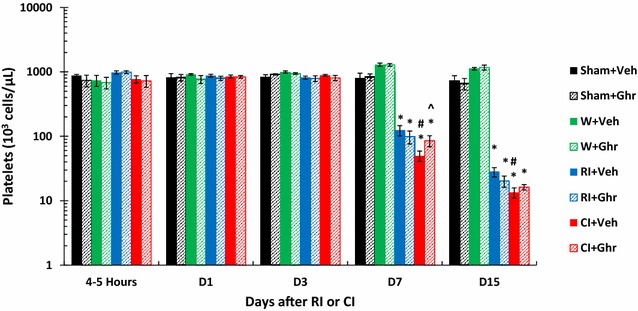
Ghrelin therapy increases platelet counts after wounding and CI. Animals were irradiated alone or followed by wounding. Blood on different time points were collected for measuring platelet counts (N = 6 per group). *p < 0.05 vs. Sham group; ^^^p < 0.05 vs. respective Veh group; ^#^p < 0.05 vs. RI + Veh group. *WBCs* white blood cells, *Veh* vehicle, *Ghr* ghrelin, *W* wounding, *RI* 9.5 Gy, *CI* 9.5 Gy + wounding

It is known that RI decreased platelets and CI further decreased them [5]. Figure 5 shows that RI-induced decreases in platelets began on day 7 and they continued to decrease on day 15. CI-induced enhancement was observed on days 7 and 15. Ghrelin therapy recovered platelet counts on days 7 and 15. Wounding alone with ghrelin increased platelet counts on days 3, 7 and 15.

### Ghrelin increases spleen weights after wounding and CI

**Fig. 6:**
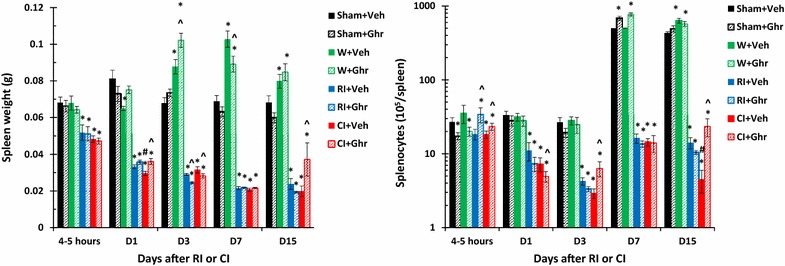
Ghrelin increases spleen weights and splenocyte counts after wounding and CI. Animals were irradiated alone or followed by wounding. Spleens on different time points were collected for measuring spleen weights and splenocyte counts (N = 6 per group). *p < 0.05 vs. Sham group; ^^^p < 0.05 vs. respective Veh group; ^#^p < 0.05 vs. RI + Veh group. *WBCs* white blood cells, *Veh* vehicle, *Ghr* ghrelin, *W* wounding, *RI* 9.5 Gy, *CI* 9.5 Gy + wounding

RI alone significantly decreases spleen weight in the beginning and then induces splenomegaly demonstrated only in surviving mice at 30 days [24]. In this study, RI began to decrease spleen weight within 4–5 h, continued to decrease on day 1 to day 7 and then remained the nadir. CI enhanced the weight reduction only on day 1. Ghrelin therapy recovered the weight on day 1 and day 15. Wounding alone with either vehicle or ghrelin significantly increased the weights on days 3, 7 and 15 (Fig. 6).

### Ghrelin increases splenocytes after wounding and CI

RI alone significantly decreases splenocyte counts within 4–5 h and continues to decrease on day 1 to day 3 then remained nadir on days 7 and 15. CI enhanced the reduction of cell counts on days 1, 3 and 15. Ghrelin therapy recovered the cell counts on days 3 and 15 in CI mice. Ghrelin significantly increased the cell counts in sham mice and wounded mice on days 7 and 15 (Fig. 6).

### Ghrelin therapy enhances and sustains G-CSF increases after CI

**Fig. 7:**
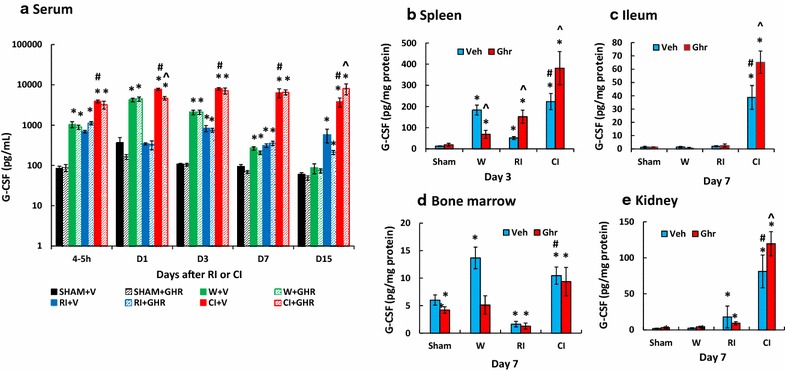
Ghrelin therapy enhances and sustains G-CSF increases after CI. Animals were irradiated alone or followed by wounding. Blood on different time points were collected for measuring G-CSF concentrations (N = 6 per group). *p < 0.05 vs. Sham group; ^^^p < 0.05 vs. respective Veh group; ^#^p < 0.05 vs. RI + Veh group. *WBCs* white blood cells, *Veh* vehicle, *Ghr* ghrelin, *W* wounding, *RI* 9.5 Gy, *CI* 9.5 Gy + wounding

RI and CI have been shown to increase G-CSF and CI increases it more than RI [4]. As shown in Fig. 7, we found that wounding increased G-CSF at 4–5 h, days 1, 3 and 7, whereas ghrelin did not enhance the increases. RI increased G-CSF at 4–5 h but returned to the baseline on day 1, then increased again on days 3, 7 and 15. Ghrelin diminished the RI-induced increases on day 15. CI enhanced its increases more than RI at 4–5 h, days 1, 3, 7 and 15. Ghrelin therapy further increased G-CSF on day 15. This ghrelin-enhanced increase was corresponded with the enhanced increase in spleen (Fig. 7b), ileum (Fig. 7c) and kidney (Fig. 7d) but not in bone marrow cells (Fig. 7e). Spleen, ileum, and kidney displayed CI-enhanced increases in G-CSF while bone marrow exhibited no CI enhancement. The data on day 3 in spleen were presented, different from day 7 in other organs, because there were no changes in G-CSF on day 7.

### Ghrelin therapy enhances KC increases after CI

**Fig. 8:**
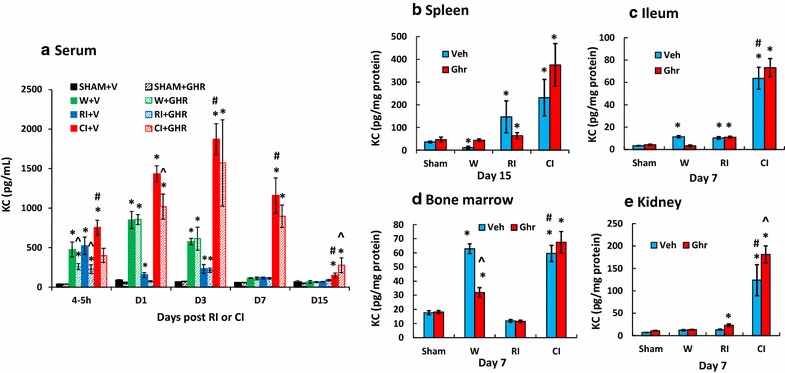
Ghrelin therapy enhances KC increases after CI. Animals were irradiated alone or followed by wounding. Blood on different time points were collected for measuring G-CSF concentrations in serum (N = 6 per group). *p < 0.05 vs. Sham group; ^^^p < 0.05 vs. respective Veh group; ^#^p < 0.05 vs. RI + Veh group. *WBCs* white blood cells, *Veh* vehicle, *Ghr* ghrelin, *W* wounding, *RI* 9.5 Gy, *CI* 9.5 Gy + wounding

RI and CI have been shown to increase KC and CI increases it more than RI [4]. As shown in Fig. 8, we found that wounding increased KC at 4–5 h, days 1, 3, and 7 and returned to the baseline on day 15, whereas ghrelin inhibited the increase at 4–5 h, but left KC alone on days 1 and 3. RI increased KC at 4–5 h. The RI-induced KC increase was diminished on day 1 and 3 and returned to the baseline on day 7. Ghrelin therapy inhibited RI-induced KC increases on 4–5 h and day 1. CI further increased KC on 4–5 h, days 1, 3, 7, and 15. Ghrelin diminished the RI-induced increases on day 15. CI enhanced its increases more than RI at 4–5 h, days 1, 3, 7 and 15. Ghrelin therapy attenuated the KC increases at 4–5 h and day 1, but enhanced the KC increase on day 15. This ghrelin-enhanced increase in blood was corresponded with the enhanced increase in KC found in kidney (Fig. 8d) but not in spleen (Fig. 8b), ileum (Fig. 8c) and bone marrow cells (Fig. 8e). Kidney displayed an enhanced increase in KC after CI while spleen, ileum and lung exhibited no KC enhancement by CI. The data at day 15 in spleen were presented, different from day 7 in other organs, because there were no changes in KC levels in spleen.

### Ghrelin therapy decreases caspase-3 after CI

**Fig. 9:**
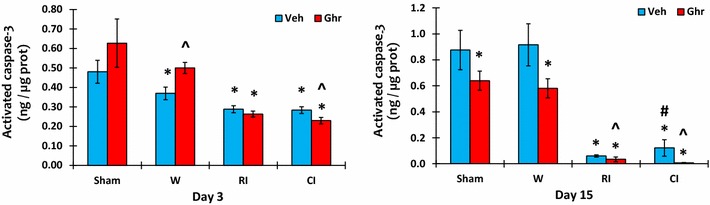
Ghrelin therapy decreases caspase-3 after CI. Animals were irradiated alone or followed by wounding. Blood on different time points were collected for measuring KC concentrations in serum (N = 6 per group). *p < 0.05 vs. Sham group; ^^^p < 0.05 vs. respective Veh group; ^#^p < 0.05 vs. RI + Veh group. *WBCs* white blood cells, *Veh* vehicle, *Ghr* ghrelin, *W* wounding, *RI* 9.5 Gy, *CI* 9.5 Gy + wounding

CI increases caspase-3 more than RI in ileum [39, 40]. In this study, we found significant decreases in caspase-3 in the bone marrow of RI and CI animals from day 1 through day 15. On day 3, ghrelin therapy significantly decreased caspase-3 levels in RI mice on day 15. In CI mice, ghrelin therapy significantly decreased caspase-3 levels on day 3 and day 15. In sham and wounded animals, ghrelin did not affect caspase-3 baselines on day 3, but significantly diminished caspase-3 levels on day 15 (Fig. 9).

## Discussion

Our novel results are the first to show that ghrelin therapy sustained G-CSF and KC increase in bone marrow and reduced bone marrow injury in CI mice. The amelioration of bone marrow injury began as early as day 1 and continued to sustain up to day 15. Perhaps because that amelioration took place at day 1 in bone marrow, peripheral WBCs of all types were better recovered on day 3, although only neutrophils and monocytes continued to increase on day 15.

Neutrophils are matured in bone marrow, resided and retained in the hematopoietic cords of bone marrow reserve, which is mediated by stromal cell-derived factor (SDF-1α) constitutively expressed in bone marrow. Neutrophils in bone marrow have chemokine receptor 4 (CXCR4) expressed on their surface. G-CSF treatment inhibits SDF-1α [41] and CXCR4 [42, 43] expressions and mobilizes mature neutrophils into circulation. The senescent neutrophils become apoptotic neutrophils. The latter is removed by bone marrow macrophages and becomes degraded in liver and spleen. This action of macrophage destroying apoptotic neutrophils stimulates the G-CSF production in bone marrow. When neutrophils are being mobilized to circulation from bone marrow reserve, they need to cross the sinusoidal endothelium. The crossing process is regulated by G-CSF [44]. Normally, approximately 10^11^ neutrophils exit the bone marrow daily. However, their half-life in the blood is 6.5 h [see review, 45]. Under episodes of inflammation or infection, neutrophils quickly exit bone marrow and are recruited to the site through chemoattraction of KC and macrophage inflammatory protein-2 (MIP-2) within a matter of hours [46].

Ghrelin, a stomach-derived peptide, has a half-life of approximately 31 min in plasma [37, 38]. In this study, we found that ghrelin therapy sustained the circulating G-CSF increase, which was significantly contributed by ileum, spleen, kidney, bone marrow and perhaps other untested organs, stimulated myeloid progenitors in bone marrow for neutrophil proliferation and maturation. Then, G-CSF disrupted the retention signal delivered by SDF-1α and facilitated the migration of neutrophils across the bone marrow sinusoidal endothelium in response to the chemotactic gradient created by blood chemokines such as KC. Neutrophil mobilization to circulation led to an increase in blood neutrophil counts that were observed and beneficial against systemic bacterial infection [4, Fig. 3], thereby, leading to increased animal survival.

RI and CI significantly reduce WBCs as previously reported [5, 7]. In the current study, by day 30 after RI or CI, surviving mice still displayed low values of WBCs, mainly neutrophils, lymphocytes, monocytes and eosinophils (Fig. 3). However, in CI mice, in addition to circulating neutrophil increases, ghrelin therapy increased other types of leukocytes, suggesting that ghrelin accelerates overall bone-marrow cell proliferation and maturation, as confirmed in Fig. 2. Ghrelin may have acted via GHSR-1α (i.e., ghrelin receptors, coupling to G protein)-mediated PI3K/Akt/eNOS/NO signal pathway [30] to initiate proliferation and differentiation of myeloid progenitors into mature granulocytes and induce hematopoietic stem-cell mobilization from the bone marrow into the bloodstream. On the other hand, because GHSR-1α is expressed in lymphocytes [47], the action of ghrelin directly to preserve lymphocytes cannot be ruled out.

RI and CI significantly reduce RBCs, hemoglobin levels, and hematocrit values as previously reported, and CI reduces more than RI [5, 7]. In the current study, the comparable results were found. On day 30 after RI or CI, surviving mice still displayed low values of RBCs (Fig. 4). Ghrelin did not mitigate RI or CI-induced decreases in RBC counts, hemoglobin levels, and hematocrit values. The results are inconsistent with the observation in mice exposed to radiation followed by burn [24]. Nevertheless, ghrelin treatment improved numbers of platelets in CI mice but not in RI-mice, a similar observation after radiation followed burn [24]. This ghrelin differential effects between RI and CI might suggest that this peptide also could stimulate megakaryocytes in the bone marrow (Fig. 1) under certain yet undefined conditions, like platelet recovery resulting from IL-12 treatment [48] and pegylated G-CSF [7]. From our results, we postulate that ghrelin passes the bone barrier and enters bone marrow to promote the thrombocytogenesis from megakaryocytes and releases platelets to peripheral blood to mitigate the platelet depletion in CI mice (Fig. 6), a previously unrecognized effect of ghrelin. This thrombocytogenesis may not be associated with thrombopoietin (TPO) because ghrelin therapy decreased TPO (data not shown).

We observed that RI and CI reduced spleen weights and splenocyte counts. Radiation is known to decrease CD4+ and CD8+ T cells and increase CD4+ CD8+ T cells in the thymus and decreases CD4+ and CD8+ T cells in the spleen [48]. We found that ghrelin mitigated the CI-induced spleen weight loss and splenocyte loss on day 3 and day 5 (Fig. 6). Ghrelin might stimulate proliferation of splenic T cells [34]. It is reported [34] that ghrelin is expressed in T cells and exerts prothymic and anti-inflammatory effects. It is segregated within the lipid raft domains upon TCR ligation. Silencing ghrelin gene in primary human T cells activates IκB and increases Th1 cytokines and IL-17 secretion [34]. Our dynamic cytokine profile in blood after RI and CI agrees with the observation in human cells (Kiang et al. unpublished data). Further studies to elucidate the mechanisms of CI-induced mitigation will be required to explain the different responses of RI and CI mice.

Caspase-3 is a critical protease in caspase-dependent apoptosis [49, 50]. It is increased in ileum after irradiation and combined injury [40]. In bone marrow, no increased activated caspase-3 levels were found, but RI and CI decreased caspase-3 levels on day 3 and continue to decrease the levels on day 15. Ghrelin therapy significantly reduced its levels in all sham, wounded, RI, and CI animals for stopping apoptosis (Fig. 9). It is evident that caspase-independent necroptosis is another form of programed cell death that involves receptor-interacting protein kinase 1 (RIPK1) and RIPK3. RIPK1 activates RIPK3 [51]. Our preliminary data indicate that ghrelin reduces RIPK3 in bone marrow on day 3 after RI and CI, suggesting presence of a RI and CI-induced RIPK-induced necroptosis. However, radiation-mediated crosstalk among apoptosis, necroptosis, and pyroptosis exists in immune cells. Therefore, pyroptosis in bone marrow cannot be ruled out and should be explored.

Ghrelin is found to reduce Bax (a pro-apoptotic protein) and increase Bcl-2 (an anti-apoptotic protein) in a chronic liver injury model [28]. Determining the differential activities of ghrelin on Bax and Bcl-2 in bone marrow of RI and CI mice would be informative, considering that mice in these models of acute injuries do not have chronic liver disease.

Ghrelin has been demonstrated as a countermeasure against radiation combined with sepsis [14]. It is suggested the effect is based on complex neurogenic effects of this peptide, involving with activation of cholinergic pathway, inhibition of sympathetic nervous system (SNS) and down-regulation of proinflammatory cytokines [52–54]. Therefore, ghrelin’s beneficial effects following irradiation combined with sepsis may have been correlated with the rebalance of the dysregulated sympathetic and parasympathetic (PNS) nervous systems [52]. It is possible that ghrelin-induced improvement of survival in our CI model is mediated by the rebalance of cytokines, SNS and PNS. This hypothesis requires confirmation.

We found that ghrelin sustained G-CSF and KC levels in bone marrow and circulatory blood (Figs. 7, 8), which promoted neutrophil proliferation, maturation and mobilization from bone marrow to circulation and injured areas. This unique feature makes ghrelin a better candidate than other G-CSF stimulators such as Toll-like receptor 2 agonists [55]. The latter induces side effects including appetite loss and edema [55]. Ghrelin administration accelerates body weight recovery and is found no edema [31].

In summary, skin wounds enhanced the RI-induced bone marrow injury as indicated by increases in adipocyte counts and decreases in megakaryocyte counts that were reversed by ghrelin therapy and confirmed by increased bone marrow cell counts. In CI mice, ghrelin therapy significantly recovered circulating WBCs and platelet counts but not RBCs. The recovery of neutrophil counts was mediated by sustained increases in G-CSF and KC in serum and spleen, ileum, lung, and kidney. In bone marrow, ghrelin therapy reduced activated caspase-3 levels after CI, suggesting cell recovery. In spleen, ghrelin therapy mitigated CI-induced spleen weight and splenocyte losses and CI-induced enhancement of G-CSF and KC increases. These results demonstrate efficacy of ghrelin as a radio-mitigator/therapy agent for CI.

## Abbreviations

AFRRI: Armed Forces Radiobiology Research Institute
CI: combined injury
G-CSF: granulocyte-colony stimulating factor
GHSR: growth hormone secretagogue receptor
i.v.: intravenous
KC: keratinocyte chemoattractant
p.o.: *per os*
RBCs: red blood cells
RCI: radiation combined injury
RI: radiation injury
s.c.: subcutaneous
TBSA: total body surface area
VSD: Veterinary Science Department
W: wound
WBCs: white blood cells
